# The Efficacy of Intra-Articular Versus Extra-Articular Corticosteroid Injections in the Thumb Carpometacarpal Joint

**DOI:** 10.1016/j.jhsg.2022.01.002

**Published:** 2022-02-11

**Authors:** Brian M. Katt, Amr M. Tawfik, Jomar Aryee, Daren Aita, Pedro K. Beredjiklian, Daniel Fletcher

**Affiliations:** ∗ Rutgers Robert Wood Johnson Medical School, New Brunswick, NJ; † Rothman Orthopaedic Institute, Philadelphia, PA

**Keywords:** Carpometacarpal joint, Corticosteroid injection, Injection accuracy, Intra-articular injection, Thumb arthritis

## Abstract

**Purpose:**

This study evaluated whether the location of steroid deposition (intra-articular vs extra-articular) for thumb carpometacarpal (CMC) joint arthritis affects clinical outcomes.

**Methods:**

We prospectively enrolled 102 hands (82 patients) with thumb CMC joint arthritis. Patients received a CMC joint injection with Triamcinolone and radiopaque contrast. Wrist radiographs were used to visualize the injection location. Patients completed Disabilities of the Arm, Shoulder, and Hand Questionnaire (DASH) questionnaires and visual analog scale (VAS; scale, 1–100) pain scores before injection and then at 1 week and 1, 3, and 6 months after injection. Generalized linear regression models were constructed to identify variables associated with clinical outcomes.

**Results:**

The rate of intra-articular injection was 80%. No differences were found between the 2 groups in preinjection DASH or VAS scores. After 1 week, both the intra-articular and extra-articular groups showed improvements of DASH (14.2 and 11.2, respectively) and VAS (15.5 and 15.0, respectively) scores. Although both groups were worse at 3 months, the intra-articular group had significantly lower DASH (26.7 vs 37.5, respectively) and VAS (26.5 vs 39.0, respectively) scores than the extra-articular group. There were no differences between the intra-articular and extra-articular groups for DASH (33.8 vs 42.5, respectively) or VAS scores at 6 months. The intra-articular group maintained significant improvements in outcomes for up to 6 months, while the extra-articular group only maintained them for up to 1 month. The Eaton-Littler classification was found to be a predictor of DASH and VAS scores at 3 and 6 months.

**Conclusions:**

Intra-articular injection in the thumb CMC joint provides significantly greater pain relief and functional improvement compared to extra-articular injection at 3 months. Inadvertent extra-articular injection is common and appears to provide short-term pain relief and functional improvement. Some patients receiving intra-articular injections continue experiencing relief for up to 6 months.

**Type of study/level of evidence:**

Therapeutic II.

Carpometacarpal (CMC) joint arthritis is among the most common manifestations of arthritis of the hand.^1^ Treatment of CMC joint arthritis ranges from physical therapy and orthosis fabrication to excision arthroplasty and soft tissue transfer or arthrodesis.^2^ When symptoms persist despite use of minimally invasive therapies like orthosis fabrication and nonsteroidal anti-inflammatory drugs, an attempted intra-articular corticosteroid injection may be utilized.

Studies have reported a largely heterogeneous response to steroid injections in the CMC joint.^3^^,^^4^ The reason for this inconsistency in symptom relief is unclear, but some suggest that the accuracy of the steroid injection may play a role.^3^ Prior studies comparing outcomes based on the accuracy of the injection in other joints have found conflicting results.^5^, ^6^, ^7^, ^8^ However, many of these studies compared image guidance to palpation-based techniques instead of evaluating injection accuracy directly. They also had important limitations, such as small cohorts, multiple joints evaluated, or the inclusion of inflammatory arthritis. In CMC joint arthritis specifically, studies have focused on accuracy rates as opposed to outcomes.^9^^,^^10^

This study aims to evaluate whether the location (intra-articular vs extra-articular) where the corticosteroid is deposited affects clinical outcome measures after CMC joint injection. We secondarily aim to investigate what patient demographics may be associated with responsiveness to corticosteroid injection therapy. We hypothesize that the injection location will not affect Disabilities of the Arm, Shoulder, and Hand (DASH) or patient-reported pain scores.

## Materials and Methods

This was a prospective, single-center study at the Rothman Orthopaedic Institute, Philadelphia, PA. Following institutional review board approval, we enrolled all consecutive patients ages 18 and older with radiographically confirmed arthritis of the first CMC joint who elected to have an intra-articular corticosteroid injection between April 2020 and October 2020. We excluded patients with allergies to iodinated contrast or shellfish. Patients were excluded from the analysis if they did not complete a preinjection survey, did not complete all postinjection surveys, or were scheduled for CMC joint arthroplasty during the study period.

### Procedures

Patient enrollment required a history and physical, as well as a standard radiographic evaluation consistent with the diagnosis of thumb CMC joint arthritis.

Patients initially received a subcutaneous 1-cc injection of 1% lidocaine over the dorsal aspect of the thumb CMC joint, followed by an attempted intra-articular injection containing 0.5 ccs of Triamcinolone and 0.5 ccs of Iohexol contrast via a 25-gauge needle. The hand to be injected was held in a semiprone position. The joint was identified by palpation, and the needle tip was inserted just dorsal or lateral to the abductor pollicis longus tendon. The contents of the syringe were injected using a sterile technique. The injections were administered by 1 of 3 fellowship-trained orthopedic hand surgeons (B.K., D.A., D.F.). No ultrasound guidance was used during this study. Following the injection, patients underwent posteroanterior, oblique, and lateral radiographs of the wrist to visualize contrast placement. We did not otherwise intervene or track use of other adjunctive treatments (eg, orthosis fabrication, oral pain medication, etc).

## Data collection

Demographic data, including age, sex, body mass index (BMI), and hand dominance, were collected prospectively from patient charts. After the injection was administered, 1 surgeon (B.K.) examined the preinjection radiographs to determine the stage of CMC joint arthritis according to the Eaton-Littler classification system.^11^ The Eaton-Littler classification was stratified into mild disease (stage I or II) or severe disease (stage III or IV).

**Figure 1:**
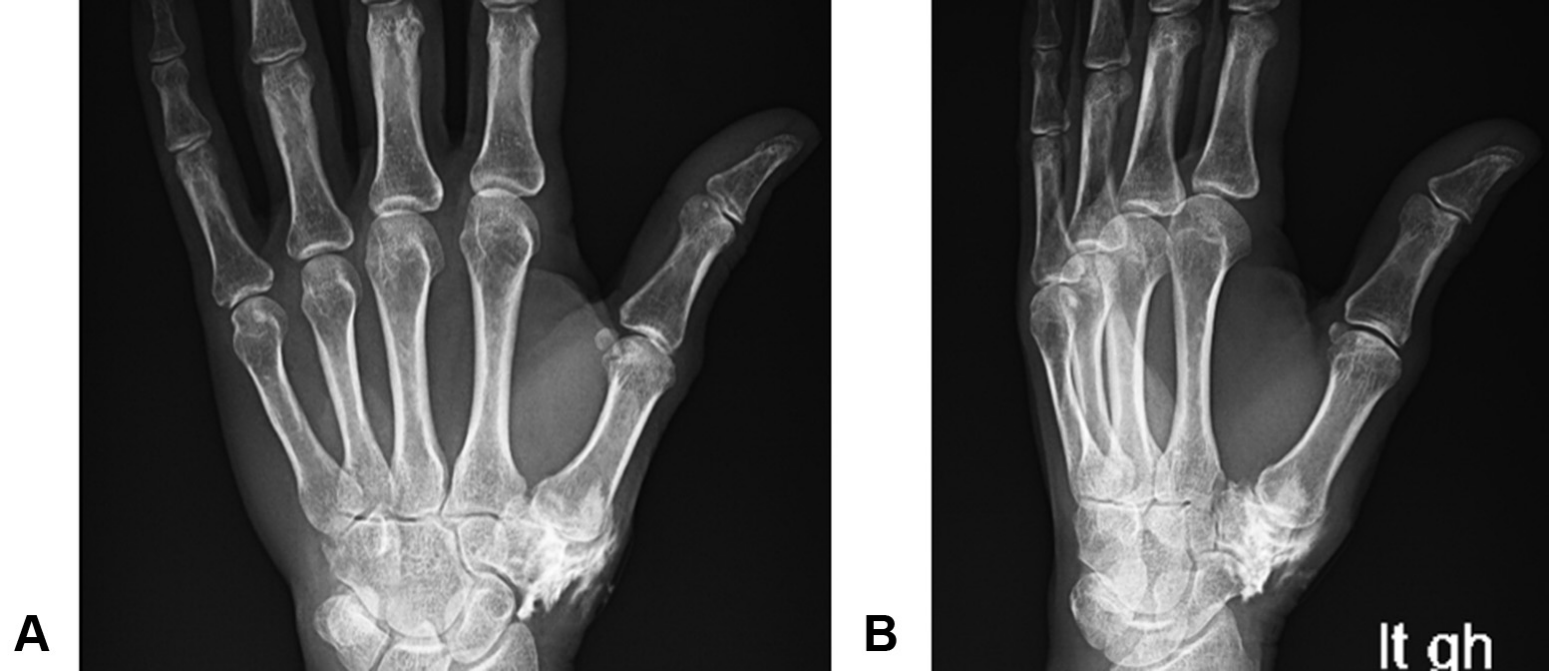
Example of an extra-articular injection, with contrast material within the surrounding soft tissue. **A** PA view. **B** Oblique view.

**Figure 2:**
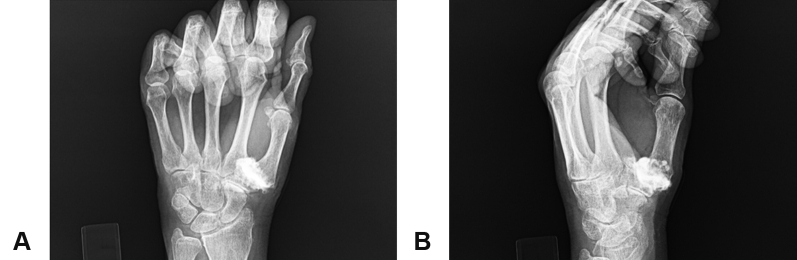
Example of an intra-articular injection, with contrast material within the joint space. **A** PA view. **B** Oblique view.

An injection was classified as intra-articular or extra-articular based on the postinjection radiographs. The presence of contrast material within only the soft tissues was classified as extra-articular (Fig. 1); otherwise, the injection was classified as intra-articular (Fig. 2). This classification was made at the conclusion of the study independently by 2 surgeons (B.K., D.F.) who were blinded to the identity of the patients. In cases where there was not agreement by the 2 surgeons, a third blinded surgeon (D.A.) was used as a tiebreaker. Patients were blinded to the results of the radiographic assessment.

The primary outcomes for this study were the DASH score and visual analog scale (VAS) patient-reported pain score. These values were recorded just before the injection and sent as a survey at 1 week, 1 month, 3 months, and 6 months following the injection. Patients undergoing bilateral injections as a part of this study were asked to answer DASH and self-reported pain questions separately for each hand. The DASH score was used to evaluate hand disability. A prior study suggests that a change in DASH score of 10.8 represents the minimum clinically important difference.^12^ Patient-reported pain was reported using a 100-mm horizontal VAS, with 0 representing no pain and 100 representing the worst pain ever experienced. It has previously been reported that for conditions of chronic joint pain, a 20% reduction in VAS scores represents a clinically significant improvement.^13^^,^^14^ The DASH and VAS scores were further plotted against time in days to graphically represent the duration of the benefit from the injections.

### Statistical methods

A sample size estimate was performed to determine that 104 hands would need to be enrolled. This was to achieve a power of 0.8 and an α of 0.05 to detect a 50% higher DASH score in the extra-articular group. This estimate was made assuming a 4:1 allocation ratio of intra-articular to extra-articular injections, based on prior estimates of extra-articular injections ranging from 18% to 37%.^9^^,^^10^^,^^15^

For data analysis, continuous data were represented by means and SDs for parametric variables and as medians and interquartile ranges for nonparametric variables. Counts and percentages were used for categorical variables. Interrater agreement on the location of steroid deposition was calculated using Fleiss’ kappa, with a value <0.5 considered weak agreement and a value >0.7 considered strong agreement. Within each group, DASH and VAS scores at each time point were compared to the baseline value using a paired *t* test. The chi-square test, paired *t* test, and Mann-Whitney U test were used to compare baseline characteristics between the intra-articular and extra-articular groups for categorical, parametric continuous, and nonparametric continuous variables, respectively. Disabilities of the Arm, Shoulder, and Hand and VAS scores at each time point were compared between the intra-articular and extra-articular groups using a Mann-Whitney U test. Clinical improvement at 3 and 6 months for both DASH and VAS scores were compared between the 2 groups using a chi-square analysis.

Two sets of generalized linear regression models were constructed using changes from preinjection values in DASH and VAS scores as the dependent outcomes. These regression models were run to identify demographic variables that may act as predictors of 3- and 6-month changes in DASH or VAS scores following injection. A *P* value < .05 was considered to be significant for all variables assessed.

## Results

During the study period, 120 hands (100 patients) were enrolled, and a total of 18 hands were excluded from the study. The reasons for exclusion include incomplete preinjection data (4; 3 intra-articular, 1 extra-articular), not having responses to all the postinjection surveys (11; 8 intra-articular, 3 extra-articular), and being scheduled for CMC joint arthroplasty during the study period (3; 2 intra-articular, 1 extra-articular). The final cohort included 102 hands (85 patients) for analysis, with 82 hands in the intra-articular group, corresponding to an injection accuracy of 80.4%. There was agreement between the 2 surgeons (B.K., D.F.) on the steroid location for all but 4 hands of the cohort. The Fleiss’ kappa score was calculated to be 0.835, indicating strong agreement between the 2 surgeons (B.K., D.F.) in their evaluation of steroid location. There were no significant differences between the groups regarding age, gender, BMI, dominant hand involvement, baseline DASH or VAS scores, or radiographic severity of thumb arthritis (Table 1).

**Table 1:** Patient Demographics

Characteristic	Intra-Articular	Extra-Articular	*P* Value
	(n = 82)	(n = 20)	
Age	64.0 ± 9.22	61.1 ± 6.66	.12
Sex (F)	63 (76.8)	17 (85.0)	.55
BMI	27.6 [24.9–31.2]	29.7 [26.5–32.6]	.14
Eaton-Littler classification			>.99
I or II	52 (63.4)	13 (65.0)	
III or IV	30 (36.6)	7 (35.0)	
Dominant hand injected	45 (54.9)	13 (65.0)	.57
Preinjection DASH	42.5 [29.4–57.3]	43.3 [36.3–48.5]	.50
Preinjection VAS	60.5 [48.0–78.8]	65.0 [52.5–72.8]	.70

Continuous variables are represented as means ± SDs for parametric variables and as medians [interquartile ranges] for nonparametric variables. Categorical variables are represented as counts (percentages of groups).

### Comparisons to baseline

For the intra-articular group, the median DASH scores at 1 week (14.2), 1 month (12.1), 3 months (26.7), and 6 months (33.8) were significantly lower than the preinjection value (42.5; *P* < .05). In the same group, the median VAS scores at 1 week (15.5), 1 month (17.5), 3 months (26.5), and 6 months (50.0) were significantly lower than the preinjection value (60.5; *P* < .05).

For the extra-articular group, the median DASH scores at 1 week (11.2) and 1 month (21.2) were significantly lower than the preinjection value (43.3; *P* < .05). Regarding the median VAS, the scores at 1 week (15.0), 1 month (31.0), and 3 months (39.0) were significantly lower than the preinjection value (65.0; *P* < .05). There were no significant differences between the preinjection and 3- or 6-month DASH or 6-month VAS scores in patients who received an extra-articular injection (*P* = .13, .28, and .37, respectively; Table 2).

**Table 2:** Comparison of Postinjection DASH Scores and VAS Pain to Preinjection Values

Score Time Period	Intra-Articular	*P* Value	Extra-Articular	*P* Value
	(n = 82)		(n = 20)	
DASH				
Preinjection	42.5 [29.4–57.3]		43.3 [36.3–48.5]	
1 week	14.2 [1.67–16.7]	<.01∗	11.2 [2.29–16.9]	<.01∗
1 month	12.1 [5.00–29.8]	<.01∗	21.2 [3.75–33.3]	<.01∗
3 months	26.7 [11.7–40.0]	<.01∗	37.5 [20.2–55.8]	.13
6 months	33.8 [22.7–42.5]	<.01∗	42.5 [29.6–47.7]	.28
VAS				
Preinjection	60.5 [48.0–78.8]		65.0 [52.5–72.8]	
1 week	15.5 [2.75–23.0]	<.01∗	15.0 [2.25–25.0]	<.01∗
1 month	17.5 [3.00–33.8]	<.01∗	31.0 [14.2–42.0]	<.01∗
3 months	26.5 [12.8–45.0]	<.01∗	39.0 [25.8–65.2]	.02∗
6 months	50.0 [38.5–69.6]	<.01∗	50.0 [47.3–74.3]	.37

Data are represented as medians [interquartile ranges].

∗ Statistically significant value at a *P* value <.05.

### Comparisons between groups

**Figure 3:**
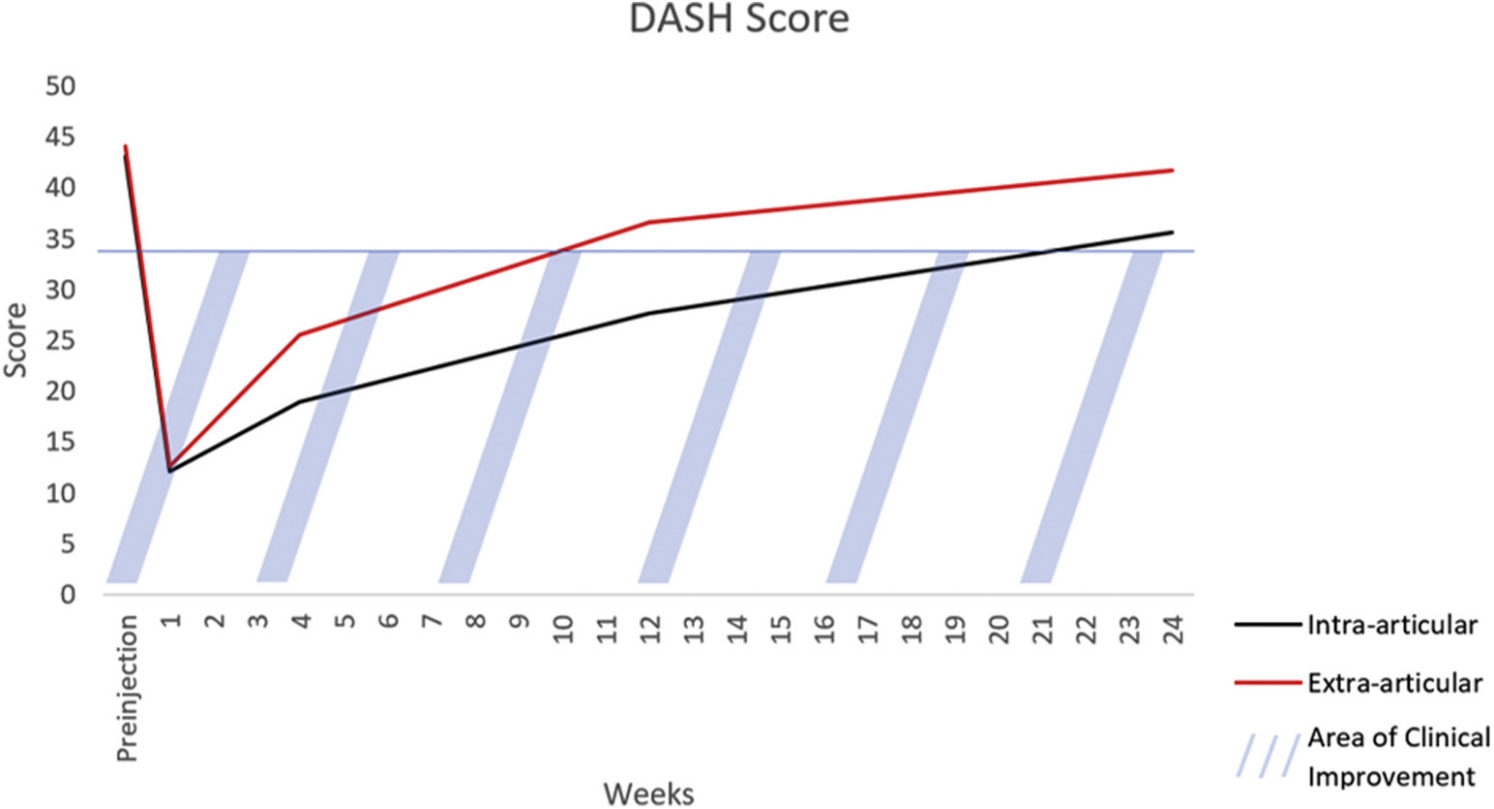
Changes in DASH score from time of injection to 6 months. Values below the blue line represent clinical improvement in DASH from preinjection DASH score.

Table 3 summarizes outcomes between the intra-articular and extra-articular groups. There were no significant differences in either DASH or VAS scores between the 2 groups at the preinjection, 1-week, or 1-month time points. The DASH score was significantly lower in the intra-articular group at 3 months compared to the extra-articular group (26.7 vs 37.5, respectively; *P* < .05). Figure 3 depicts the time until DASH scores returned to baseline, on average.

**Table 3:** Comparison of DASH Scores and VAS Pain Between Groups Following CMC Steroid Injections

Score Time Period	Intra-Articular	Extra-Articular	*P* Value
	(n = 82)	(n = 20)	
DASH			
Preinjection	42.5 [29.4–57.3]	43.3 [36.3–48.5]	.50
1 week	14.2 [1.67–16.7]	11.2 [2.29–16.9]	.88
1 month	12.1 [5.00–29.8]	21.2 [3.75–33.3]	.52
3 months	26.7 [11.7–40.0]	37.5 [20.2–55.8]	.04∗
6 months	33.8 [22.7–42.5]	42.5 [29.6–47.7]	.12
VAS			
Preinjection	60.5 [48.0–78.8]	65.0 [52.5–72.8]	.70
1 week	15.5 [2.75–23.0]	15.0 [2.25–25.0]	.87
1 month	17.5 [3.00–33.8]	31.0 [14.2–42.0]	.17
3 months	26.5 [12.8–45.0]	39.0 [25.8–65.2]	.03∗
6 months	50.0 [38.5–69.5]	50.0 [47.3–74.3]	.40

Data are represented as medians [first quartile–third quartile].

∗ Statistically significant at a *P* value <.05.

**Figure 4:**
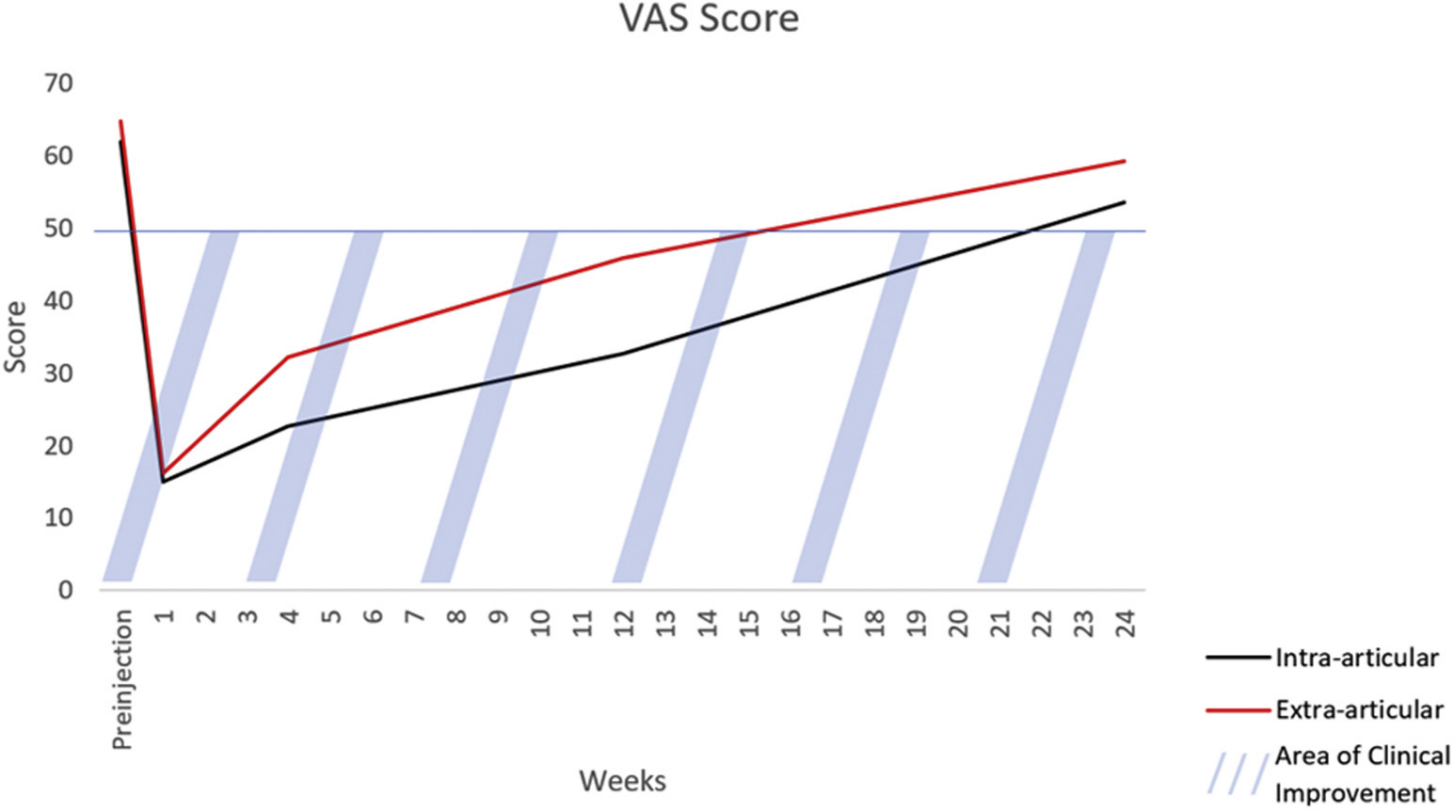
Changes in VAS score from time of injection to 6 months. Values below the blue line represent clinical improvement in VAS from preinjection VAS score.

Similarly, the VAS score was significantly lower at 3 months for the intra-articular group compared to the extra-articular group (26.5 vs 39.0, respectively; *P* < .05). At 6 months after injection, neither the DASH (33.8 vs 42.5, respectively; *P* = .12) nor VAS (50 in both groups; *P* = .4) scores significantly differed between the intra-articular and extra-articular groups. Figure 4 depicts the time until VAS scores returned to baseline, on average.

### Regression analysis

Tables 4 and 5 summarize factors associated with changes in DASH and VAS scores at 3 and 6 months after injection, respectively. Age, sex, BMI, affected hand, and hand dominance were not found to be significant factors for changes in DASH or VAS scores at 3 or 6 months. Severe arthritis (Eaton-Littler classification III or IV) was associated with increased DASH scores at 3 months after injection (*P* < .05). Additionally, intra-articular injection was associated with lower DASH and VAS scores at 3 months after injection (*P* < .05). At 6 months after injection, severe arthritis was associated with higher DASH and VAS scores (*P* < .05), but injection placement was not.

**Table 4:** Factors Affecting DASH and VAS at 3 Months After Injection ∗

Predictors	3-Month Delta DASH			3-Month Delta VAS		
	Estimates	CI	*P* Value	Estimates	CI	*P* Value
Age	0.16	-0.28 to 0.61	.47	0.52	-0.19 to 1.22	.15
Sex†	8.10	-1.31 to 17.51	.10	-2.60	-17.53 to 12.34	.73
BMI	-0.55	-1.25 to 0.14	.12	-0.57	-1.67 to 0.54	.32
Dominant Hand‡	-3.63	-11.04 to 3.78	.34	-2.46	-14.22 to 9.29	.68
Injection Location§	-8.24	-13.06 to -3.42	.01‖	-11.40	-21.70 to -1.10	.04‖
Eaton-Littler score¶	20.92	13.18 to 28.66	<.01‖	11.18	-1.11 to 23.46	.08

∗ A generalized linear regression model with delta DASH and VAS at 3 months after injection was used as the dependent outcome.

† Female sex was used as the reference group.

‡ Nondominant hand was used as the reference group.

§ Extra-articular steroid deposition was used as the reference group.

‖ Statistically significant at a *P* value <.05.

¶ Mild disease (stage I or II) was used as the reference group. The R^2^ Nagelkerke was 1.000 for all estimates.

**Table 5:** Factors Affecting DASH and VAS at 6 Months After Injection ∗

Predictors	6-Month Delta DASH			6-Month Delta VAS		
	Estimates	CI	*P* Value	Estimates	CI	*P* Value
Age	0.02	-0.38 to 0.42	.93	0.43	-0.17 to 1.04	.17
Sex†	-1.23	-9.76 to 7.29	.78	-3.29	-16.13 to 9.56	.62
BMI	-0.45	-1.08 to 0.18	.17	-0.12	-1.07 to 0.83	.80
Dominant hand‡	-6.09	-12.80 to 0.62	.08	-4.34	-14.45 to 5.77	.40
Injection location§	-4.67	-13.13 to 3.79	.28	-4.70	-17.45 to 8.05	.47
Eaton-Littler score‖	9.34	2.33–16.35	.01¶	12.37	1.80–22.93	.02¶

∗ A generalized linear regression model with delta DASH and VAS at 6 months after injection was used as the dependent outcome.

† Female sex was used as the reference group.

‡ Nondominant hand was used as the reference group.

§ Extra-articular steroid deposition was used as the reference group.

‖ Mild disease (stage I or II) was used as the reference group. The R^2^ Nagelkerke was 1.000 for all estimates.

¶ Statistically significant at a *P* value <.05.

## Discussion

The purpose of our study was to evaluate whether the location where corticosteroid is deposited affects patient-reported outcome measures for patients with thumb CMC joint arthritis. Our data support that the effectiveness of corticosteroid injection therapy for thumb CMC joint arthritis depends on the location where the steroid is deposited (intra-articular vs extra-articular). Patients who received inadvertent extra-articular corticosteroid injections reported improvements in pain and function compared to baseline that decreased by 3 months. Patients who received intra-articular injections reported improvements in both pain and function lasting through the 3-month period but returning to baseline by 6 months. Notably, 3 months after injection was the last time point at which patients who received intra-articular injections reported significantly better pain and function scores when compared to those who received an extra-articular injection. The 3-month time point was also the last time that a significantly greater number of patients in the intra-articular group than the extra-articular group reported clinically meaningful improvements in DASH or VAS scores. This seems to suggest that intra-articular injections lead to an increased duration of pain relief and functional improvement; however, by 6 months the benefits of all injections have diminished, similar to what has been described in prior studies.^3^ This knowledge helps to clarify the narrative surrounding inconsistent medium-duration responses to corticosteroid injection therapy by providing a reason why some patients may report more significant and prolonged symptom relief than others. Moreover, our findings that intra-articular injection at the thumb CMC joint improves pain and function compared to baseline for up to 6 months (but trends back to baseline) helps treating physicians provide a more accurate prognosis for their patients.

The positive effects of intra-articular steroid injection were recognized as early as the mid-20^th^ century.^16^ The use of intra-articular steroid injection for the treatment of osteoarthritis is common practice in multiple joints, but the utility and toxicity of the therapy are not entirely understood.Click or tap here to enter text.^17^ For thumb CMC joint arthritis specifically, previous literature has found the pain relief to be short term.^3^^,^^4^^,^^17^ However, the specific amounts and durations of pain relief vary widely in the literature. In a recent systematic review and meta-analysis, Riley et al^18^ compared the results of 9 randomized control trials that examined the effectiveness of corticosteroid injection therapy for thumb CMC joint arthritis. Ultimately, the authors were unable draw a conclusion on the benefits of injection-based therapy in treating CMC joint arthritis. In another systematic review, Fowler et al^3^ compared 9 studies, including 5 randomized control trials. They found the efficacy of steroid injections to vary from no benefit compared to placebo to significant benefits at 6 months. This wide variation in success is characteristic of the literature, and Fowler et al^3^ postulate that inaccurate steroid deposition is a factor that may affect outcomes. Our data stand in support of that theory, demonstrating that accurate injection of the CMC joint resulted in better outcomes at 3 months. For this reason, we recommend considering the use of image guidance, such as ultrasound, when consistent intra-articular injections cannot otherwise be achieved. Image guidance has been shown to increase the accuracy of intra-articular injections in the CMC joint, with rates around 94%.^10^^,^^19^ The relatively low cost and lack of ionizing radiation make ultrasound a potentially useful tool for increasing injection accuracy in the office setting.

A noteworthy finding in our study is that patients appreciated improvements from baseline pain and function for up to 3 months when the steroid was deposited outside the joint. There are several possible reasons for such a finding. Steroids may have a local anti-inflammatory effect on the joint capsule and surrounding tissues, providing the nonsustained pain relief observed in the patients who had extra-articular steroid deposition. The systemic effects of corticosteroid injections and their impacts on osteoarthritic joint disease are presently being studied in clinical trials.^20^^,^^21^ Wang et al,^22^ in their 2021 multicenter randomized controlled trial, found that gluteal glucocorticoid injections are not inferior to intra-articular knee injections for reducing knee pain in osteoarthritis at 8 and 24 weeks. While the exact mechanism has not yet been defined, evidence for the effects of systemic corticosteroid use on joint osteoarthritis is emerging. That effect may have played a role in the results observed in this study. It is also plausible that in injections that we categorized as extra-articular, a small amount of steroid did deposit within the joint, contributing to the short-term relief patients experienced. This is not surprising, as Pollard et al^9^ previously described contrast extravasation as a common occurrence following injections.

We also observed that the severity of arthritis, based on the Eaton-Littler classification, was associated with treatment outcomes following injection. Most patients with grade III or IV arthritis in both groups did not derive as much relief as those with grade I or II arthritis, as measured by DASH or VAS at 3 or 6 months after injection. This finding is similar to the conclusion made by Ostergaard et al,^23^ where advanced radiographic arthritis (Eaton-Littler grade III or IV) was a patient-specific factor predicting progression to surgery following injection. Prior studies support this finding, demonstrating that patients with minimal arthritic changes on plain radiographs derive more benefit and benefit for a longer duration when compared to those with more severe arthritic changes.^24^ Day et al^25^ further reported that in patients with Eaton-Littler radiographic stage I osteoarthritis of the thumb CMC joint, indicating mild disease, steroid injections combined with orthosis fabrication provided reliable pain relief, while considerably fewer patients with stages II and III disease derived longer-term benefit. Day et al^25^ additionally reported that none of their patients with stage IV disease derived benefits from the injection at either short- or long-term time points.^26^ Additionally, Meenagh et al^27^ found no clinical benefit derived from steroid injections in the CMC joint of the thumb in patients with moderate to severe osteoarthritis. Considering that arthrosis may reduce the accuracy of thumb CMC joint injection and that extra-articular deposition of steroid yields less and shorter pain and dysfunction relief, it is possible that patients with high-grade arthritis are more prone to extra-articular steroid injection and, thus, less benefit from thumb CMC joint steroid injection.^10^^,^^24^^,^^25^^,^^27^ We did not observe a difference in the rate of extra-articular steroid deposition based on the severity of arthritis; however, this study was underpowered to make that specific determination. More investigation is needed to clarify that relationship.

Our study is not without its limitations. Like many others on this topic, our investigation did not consider concomitant therapies (orthosis fabrication, nonsteroidal anti-inflammatory drugs) that our patients may have used. Future studies on this topic should strive to control for this and give readers a better understanding of the effects these adjunctive therapies have on the efficacy of steroid injections. Additionally, our criteria for intra-articular injection included any amount of steroid within the joint space. Therefore, it is unclear what quantity of steroid was deposited within the joint space in our study, and that could have impacted results. The determination of injection location was made based on the evaluation of radiopaque contrast on an x-ray taken during the same office visit, instead of direct fluoroscopy. It is possible that from the time of injection to the x-ray, the contrast material may have moved or extravasated. However, we believe that any change was minimal, as the x-rays were taken shortly after the injection. Finally, our study did not evaluate the techniques or experiences of the physicians (B.K., D.F., D.A.) providing injections. However, since our rate of inaccurate joint injection was similar to what has been reported previously, we believe that this did not substantially impact the generalizability of our study.

In conclusion, extra-articular corticosteroid deposition is a common outcome for thumb CMC joint arthritis injections. By 3 months, pain and functional improvement return to baseline when there is inadvertent extra-articular deposition, and a significantly greater number of patients experience clinical improvement in pain and function at 3 months when the steroid achieves intra-articular placement. Additionally, a subset of patients may benefit from improved pain and function for up to 6 months if the injection results in an intra-articular deposit of corticosteroid. Clinicians who use this therapy should consider these factors to optimize patient outcomes.
